# A Pilot Study to Examine the Feasibility and Acceptability of a Virtual Adaptation of an In-Person Adolescent Diabetes Prevention Program

**DOI:** 10.3390/ijerph191912286

**Published:** 2022-09-27

**Authors:** Sumaiya Islam, Cordelia Elaiho, Guedy Arniella, Sheydgi Rivera, Nita Vangeepuram

**Affiliations:** 1 School of Medicine, City University of New York (CUNY), New York, NY 10031, USA; 2 Department of Pediatrics, Icahn School of Medicine at Mount Sinai, New York, NY 10029, USA; 3 Institute for Family Health, New York, NY 10035, USA; 4 Teen HEED Community Action Board, Icahn School of Medicine at Mount Sinai, New York, NY 10029, USA; 5 Department of Population Health Science and Policy, Icahn School of Medicine at Mount Sinai, New York, NY 10029, USA; 6 Institute for Health Equity Research, Icahn School of Medicine at Mount Sinai, New York, NY 10029, USA

**Keywords:** adolescent, diabetes prevention, technology, virtual delivery, peer education

## Abstract

Background: Rates of prediabetes and type 2 diabetes are alarmingly high among racial/ethnic minority youth. The current study examines the virtual adaptation of an in-person peer-led youth diabetes prevention program. Methods: The initial phase involved the study team adapting workshop sessions from an in-person to a virtual format (Zoom). We conducted a 2-h feasibility pilot in December 2020 and implemented the full 12 session pilot program from June to September 2021 with 14 prediabetic adolescents recruited from our hospital-based general pediatric clinic. Weekly sessions were led by trained peer educators and focused on promoting healthy eating and physical activity using behavioral techniques (e.g., goal setting, brainstorming, and problem solving). Results: The virtual adaptation of our program was shown to be feasible and acceptable among our pilot participants. We were able to deliver the same workshop content and behavioral skills development as the in-person workshop using a variety of Zoom features. Conclusions: Our peer-led youth diabetes prevention program was successfully adapted and implemented in a virtual format and was well accepted by at-risk youth. Future research is needed to examine the impact of virtual youth lifestyle interventions on behavioral and clinical outcomes such as weight and diabetes risk.

## 1. Introduction

Diabetes mellitus (DM) is a growing concern in the United States, with 34 million American adults having diabetes and an additional 88 million having prediabetes [1]. There has also been a rise in new diagnoses of type 1 and type 2 diabetes among youth, with a higher incidence of type 2 diabetes and prediabetes among racial and ethnic minority youth [2,3,4]. Currently, approximately one in five adolescents in the United States are affected by prediabetes [5]. Several interventions, mostly targeting adults, have been implemented to prevent or delay the progression of prediabetes to diabetes, including peer education lifestyle modification programs [6,7,8,9,10,11]. Given that unhealthy dietary behaviors and inadequate physical activity, and determinants of these behaviors such as knowledge, attitudes, beliefs, and social influences, play a large role in the development of type 2 diabetes, prevention programs should target these behaviors and behavioral determinants [12,13].

There has been a recent shift to virtual delivery of lifestyle modification interventions due to challenges such as space restrictions, need to travel, cost, and conflicting demands that make in-person educational programs difficult to deliver and sustain [14,15,16,17]. In addition, with the onset of the COVID-19 pandemic, there have been more barriers to the implementation of the healthy lifestyle behaviors emphasized in these programs, including limited access to physical activity and healthy food [18]. Furthermore, pandemic-related quarantine and social distancing requirements have accelerated the need to deliver care virtually [19,20]. Currently, the main modalities through which virtual interventions have been implemented for obesity and diabetes management include mobile health (mHealth) platforms such as mobile apps, text messaging, social media, and web-based educational programs [21,22,23,24,25,26]. An emerging form of virtual program delivery is through the use of videoconferencing platforms [27,28,29,30,31,32,33,34,35,36,37].

Virtual delivery of adult diabetes prevention programs has been shown to increase patient activation and self-efficacy and provide pivotal access to informational and emotional support [38,39]. Individuals participating in virtual programs have been shown to have higher adherence to diet and physical activity recommendations and to improve weight-related outcomes and hemoglobin A1c [38,40,41,42,43,44,45,46,47,48,49,50]. Furthermore, participants enrolled in diabetes-focused web interventions have been shown to have fewer hypoglycemic symptoms, an increase in medication adherence, and a decrease in depression symptoms [44,51].

A few studies to date have focused on virtual interventions among youth. However, the vast majority of such studies have focused on adherence to medication or other treatments. For example, studies show that adolescents with type 1 diabetes participating in web-based interventions are more willing to comply with their regimented treatment protocols [52]. There are also online support website interventions with youth living with type 1 diabetes [53]. Studies have also been conducted in children with other chronic diseases, including respiratory conditions such as asthma, traumatic brain injury, recurrent pain/headaches, and cancer [54,55]. However, to date, there is a lack of studies in youth focusing on virtual lifestyle modification programs for treatment of obesity or prevention of related conditions such as type 2 diabetes.

One strategy for implementation of youth lifestyle interventions is to use trained peer educators. Studies have shown that in-person youth peer education lifestyle interventions positively impact diet and physical activity behaviors and weight-related clinical outcomes [56]. The identification and selection of peer educators is often based on qualifications such as age, education, and merit-based accomplishments, and study teams often conduct formal training of the peer educators and provide additional support and feedback during program delivery to ensure fidelity of the intervention delivery. However, despite the effectiveness of youth peer-led lifestyle interventions, we could not find studies focused on virtual delivery of such programs.

The current study describes an adaptation of an in-person youth peer education diabetes prevention program for virtual delivery. The in-person youth program, Project Teen HEED (**H**elp **E**ducate to **E**liminate **D**iabetes), built on the work of Project HEED, a community-based group peer-led diabetes prevention workshop for prediabetic adults [6]. The adult study informed the design and some of the partnerships for the adolescent study conducted in the same community (East Harlem, New York City, NY, USA). East Harlem is a diverse neighborhood in New York City (about 43% of its residents identify as Hispanic/Latinx and 36% of its residents identify as Black [57]) with a diabetes rate more than double that of Manhattan as a whole [58]. We adapted the adult HEED program with community youth leaders, input from focus groups conducted with adolescents from the community, and a conceptual model for youth diabetes risk reduction that we collaboratively developed with community partners based on tenets of social cognitive theory, the health belief model, and the social ecological model. With our Community Action Board (CAB), we recruited prediabetic adolescents for a 12-week in-person peer-led workshop focused on nutrition label reading, portion control, group exercise, and other related topics using interactive strategies such as games, brainstorming, problem-solving, role playing, and setting weekly goals. In addition to individual level factors contributing to obesity and diabetes risk, the intervention also focuses on social, economic, and environmental factors impacting lifestyle and health [59], such as access to and affordability of healthy foods and opportunities for physical activity in the local neighborhood and social pressures to engage in unhealthy behaviors. Preliminary outcomes for a pilot version of the in-person program can be found in our previously published study [12], and the manuscript describing clinical outcomes from a randomized controlled trial of the in-person intervention is in process. This manuscript outlines the iterative process we used with our community partners to adapt the program for virtual delivery and includes preliminary qualitative data about the feasibility and acceptability of this approach among diverse prediabetic adolescents.

## 2. Methods

We describe the phases of our virtual adaptation process and qualitative assessment of the feasibility and acceptability of virtual intervention implementation below.

### 2.1. Conversion of Curriculum to Virtual Format

From July to November 2020, the study team reviewed the 12-session in-person workshop curriculum and brainstormed adaptations for virtual delivery. We reviewed multiple options for remote delivery, including those that provided the desired features and functionality and/or were used most commonly by youth in our area, including Zoom, Microsoft Teams, Google Classroom, Google Meet, Webex, Skype, and Prezi Video. We considered the types of devices participants might use to access the program (tablet/iPad, phone, and laptop) and how the chosen virtual platform and our adaptations might affect the user experience. We spoke to youth, including members of our board, about which platform they would be most comfortable with. We ultimately chose to use the HIPAA-compliant version of Zoom to ensure that the platform incorporates all necessary security controls to meet HIPAA requirements. The Health Insurance Portability and Accountability Act of 1996 (HIPAA) is a federal law that required the creation of national standards to protect sensitive patient health information from being disclosed without the patient’s consent or knowledge. While our program does not involve any sensitive or private health information being shared, we wanted to take every precaution to use a secure platform in case any such information was inadvertently shared. Our ethics review board also recommended the use of a platform that complies with such security features, and the HIPAA-compliant Zoom was the only one that had these features at the time. 

### 2.2. Feasibility Pilot

To initially assess the feasibility of remote program delivery and the acceptability of the adaptations made, we conducted a two-hour Zoom session in December 2020 with previous study participants and members of our CAB technology subcommittee. The session was facilitated by the study coordinator and a peer leader who had been trained to deliver the in-person workshop. In preparation for the pilot session, the study team reviewed the agendas and activities included in the 12 sessions and shortlisted activities to include based on tasks that are repeated throughout the 12 weeks (i.e., brainstorming, problem solving, nutrition label reading, making a weekly goal, and group exercise) and session-specific activities, such as games that required extensive adaptations and use of specific Zoom features (i.e., chat box, annotation, share screen, and breakout rooms). After determining the initial feasibility of the virtual platform, we made line-by-line changes to the curriculum from December 2020 to August 2021.

### 2.3. Virtual Teen HEED Pilot—Summer Cohort

We recruited youth peer leaders through our institution’s volunteer department and vetted applicants through written responses to questions about their prior experience and motivations for pursuing the opportunity, as well as individual video interviews. Peer leader training included two 2-h virtual sessions in which we focused on team building, group facilitation, and review of the workshop curriculum, including features on Zoom to be employed during the workshop. We met with leaders weekly to prepare for each session and debriefed with them after each session to provide feedback and troubleshoot any issues that came up.

Since we conducted a qualitative pilot study, we determined the sample size based on a systematic review for sample size determination for qualitative research [60]. This review concluded that such studies often reach saturation (no new themes emerging from ongoing data analysis, the typical standard used to determine sample sizes for qualitative studies) within a narrow range of interviews. We ultimately recruited a cohort of 14 participants to pilot the complete 12-session virtual Teen HEED program (90-min Zoom sessions held once weekly in the evening) from June to September 2021. We identified potential participants via our electronic health record system. Eligibility criteria included a hemoglobin A1c level in the prediabetic range (5.7–6.4%) within 3 months of the pilot start date, a BMI greater than the 85th percentile based on 2000 Centers for Disease Control growth curves [61], and not being a candidate for gastric bypass surgery or on metformin or other medications that can impact weight or glucose levels. Our study received ethical approval from the Program for the Protection of Human Subjects at the Icahn School of Medicine at Mount Sinai (study number 14-00359). For eligible participants aged 13–17, the study team obtained signed informed e-consent from a parent or other legal guardian. For eligible participants aged 18–21, the study team obtained signed informed e-consent from the participant. Participants consented to attending the 12-week pilot program via Zoom and to being recorded for evaluation purposes. Within a few weeks after completion of the program (post intervention), we conducted individual semi-structured interviews to obtain feedback about the workshop, and participants received a gift card and an achievement certificate.

### 2.4. Interviews and Recordings

The study team created an interview guide with ten main questions, each with three to five follow-up probing questions. We asked participants for general feedback about each activity, including how much they liked the activity on a 5-point Likert scale (1 being not at all, 3 being neutral, and 5 being a lot), feedback about virtual delivery via Zoom, thoughts about the peer leaders, and perceived helpfulness of the program in assisting participants with making healthy decisions about diet and physical activity. We conducted all interviews on Zoom in September 2021 and reviewed recordings to abstract relevant quotations.

## 3. Results

### 3.1. Creation of Virtual Teen HEED

The curriculum includes several activities that are repeated throughout the 12 sessions: brainstorming, setting weekly goals, problem solving, nutrition label reading, and group exercise. For brainstorming, peer leaders share a question/prompt related to healthy eating, physical activity, and barriers to healthy lifestyle (stress, difficult emotions, school/work schedules, family pressures, and others). Participants are invited to share their thoughts with the group in an open discussion, with ideas documented and restated at the end of the brainstorm and additional suggestions shared if not mentioned by the group. Participants also make healthy lifestyle goals to complete over the week following the workshop session with prompts to assist the participants with making their goal as specific as possible. Problem solving expands on brainstorming and goal setting to share ideas for addressing the challenges mentioned for engaging in healthy lifestyle behaviors or achieving weekly goals. Nutrition label reading involves answering questions about the most important parts of nutrition labels for making healthy choices. “Let’s Get Moving!” is a 15-min group exercise activity that provides participants with examples of simple, free/low-cost strength training, aerobic, and flexibility exercises that they can do with limited equipment and in small spaces (an important consideration for youth in our community), and modified versions of the exercises to accommodate different levels. Table 1 shows the adaptations made to major workshop activities.

### 3.2. Feasibility Pilot Session

Five previous study participants (four who had attended the in-person workshop and one who was in the control group and had not been exposed to the in-person workshop) and five members of our CAB technology subcommittee participated in a one-time pilot session to assess the feasibility and acceptability of the virtual adaptations we made. Participants ranged in age from 18–30 years and included 8 females and 2 males. The session lasted for about 90 min followed by 30 min of feedback from the group. The study team and participants discussed each activity, including adjustments from the in-person to virtual format and suggestions to make the transition to Zoom easier and more engaging. Overall, participants agreed that most games and activities felt similar to the in-person workshop due to the use of breakout rooms and the chat function on Zoom. For example, one participant remarked:

“I think the breakout rooms were great. It gave you that feeling, I remember in person we did split up into groups and talk about it. So, I think the breakout rooms work great.”

While both participants and the peer leaders felt that the chat box was a good way to keep track of who answered questions first in the games, during the pilot, we discovered that using ‘me’ in the chat for every question was often confusing, as one participant remarked:

“…I thought it was kind of annoying and it’s very inconvenient because you don’t really know who said ‘me’ first. Unless somebody is keeping strict tabs on who said ‘me’ first, it’s gonna get confusing. So maybe each question is a different number and whoever types that number first is the equivalent to saying ‘me’ first. Or you could just write out your name…”

Similarly, one of the peer leaders commented,

“Maybe what we could do is use a word pertaining to the activity, so like ‘soda’ or ‘orange’ or the flavor of something for that specific activity. ‘Cause even as a peer leader looking on I’d have to check the time of when the response was given because I would look at 8 different ‘me(s)’ from everybody’s response and it can get a little bit confusing for the peer leader looking because I have to navigate between the PowerPoint, looking at you guys, and then looking at the chat, and manual.”

Due to this discussion, we decided to have participants type in a key word based on the question being asked (e.g., portion, serving, etc.) or directly type the answer to the question in the chat.

We also assessed the feasibility of adapting popular interactive team-based games used in the in-person workshop for Zoom. For example, for our version of Pictionary, the peer leader sent a private message to participants in the chat with a word describing strategies to avoid unhealthy eating when dealing with difficult emotions. Participants used the ‘annotate’ function on Zoom to draw, type, and stamp images on a shared whiteboard screen so that their teammates could guess the word.

For our version of Family Feud, we split the group into two teams and asked them to name the top six benefits of exercise, barriers/challenges to eating healthy, and ways to control portion sizes. We shared PowerPoint slides for each question and used the ‘annotate’ function on Zoom to document answers on our list and keep track of points. Participants remarked:

“On the Family Feud I think it’s really interesting because we got to work as a team as opposed to what most colleges do which is do a Kahoot and quiz people on information.”

Another activity we included in our feasibility pilot session was our group exercise demonstration and practice, ‘Let’s Get Moving’. For this activity, we used screen share to play a premade exercise video (including a warm-up, three different exercises with modifications, and a cool down with music) and asked participants to follow along. Participants discussed how conducting this activity on Zoom was different from the in-person workshop and their general comfort with conducting group exercise on Zoom. Participants also remarked on the potential benefits and challenges of doing exercise in their home. Participants stated:

“Doing the exercises in-person during the program there were yoga mats, and everything was prepared…everything was already available there for you to do the exercise so it was pretty easy. However, if you’re doing it on Zoom, then maybe you can try to accommodate the space, but not everyone has the space to layout, especially exercises you have to be on the floor for—that could be a bit difficult because maybe they can’t go to a more spacious area because their siblings are there or their parents are there and making a lot of noise as well.”

“To me it actually feels a little less awkward only because in-person everybody’s there, you can literally look at everybody right there, but on zoom you don’t notice typically because everybody is on a small screen at the top when you’re sharing the screen with the video. So, you’re not really seeing anybody else unless you’re actually looking up to the screen. So, I think it’s a bit better in terms of not wanting people to see you actually do the exercise.”

“It’s very hard to see each other so it’s very easy if you wanted to stop and not continue to do the exercises, you could get away with that, especially since there’s no one to see you.”

For the last activity of the virtual feasibility pilot session, we asked everyone to make and share a goal that they would complete over the next week. On Zoom, we discussed using the breakout room function to create small groups for making goals but also wanted to test the feasibility of doing this activity in the larger group.

Finally, we asked if teenagers would participate in a virtual version of Teen HEED and received overall positive feedback. Participants stated:

“Yes, I think [teens would come and do this online program] because it is very interactive and maybe now more than ever kids will want to interact not in a school setting.” (Referencing the social distancing measures due to the COVID-19 pandemic)

“I definitely think it would be a lot more convenient too. There were instances in the in-person program where some people couldn’t make it or lived too far, so I guess there would be a lot less of those types of instances, so most likely more people would show up. But there’s also the barrier of internet connection.”

### 3.3. Pilot of 12-Week Virtual Teen HEED Program

We initially generated a list of 125 potential participants by creating a report from our electronic health record system based on the eligibility criteria we described, including BMI in the overweight/obese range and age 13–19. We then identified 49 patients from this list who had a hemoglobin A1c in the prediabetes range within the previous 3 months. We conducted a brief chart review to ensure that patients were not on medications such as metformin, which can impact weight and glucose levels, and did not have diagnoses such as serious medical or psychiatric conditions, autism, or developmental delay that would make it difficult for them to participate in the virtual workshop. We reached out to all the remaining eligible patients (18–19 years) or caregivers of patients < 18 years (41 individuals) to inform them about the study and assess interest and availability for participation. We ultimately identified 14 adolescents who were eligible and enrolled in the study (Table 2; 36% female, 64% male, 29% Black, 64% Latino/a, mean (sd) age 14.6 (1.8) years); 50% of those consented remained engaged over the full 12 weeks (5 males and 2 females). We reached five of the seven fully engaged participants to obtain feedback during individual interviews and reached theme saturation with this sample size.

### 3.4. Overall Participant Feedback

In general, participants remarked that the virtual Teen HEED program was advantageous and applicable to their lives, giving them a greater understanding of healthy eating and active living. For example, one participant stated:

“Healthy—portion planning, healthy plating, when I was at the doctor last Thursday, I lost a couple pounds.”

“Yes. I have been trying to get fit and I didn’t know a lot about diet until I came here. I don’t eat as much as I normally do. I started looking at the food labels. Very informative so I know what to do…”

When participants were asked for adjectives to describe the program, the top words were fun and helpful. One participant stated:

“Thankful, helpful, grateful. I’m very grateful for this program to meet all of you and be part of something. It was not a waste of time. I would definitely do it [again].”

### 3.5. Peer Leaders

A significant part of the program is the use of peer leaders, young people close in age to the participants. Participants were asked to rate the peer leaders and discussed what the study team should look for when choosing future peer leaders. Some stated:

“They all get 5/5. The peer leaders were consistent with trying to keep us on our goals. It makes us feel better that someone is looking out for us. The peer leaders were funny. They don’t need to improve. The class had a super cool vibe. Look for vibrant, enthusiastic, fun, and happy people who radiate energy even if I’m not feeling it that day.”

### 3.6. General Comments about the Virtual Experience

The virtual Teen HEED program allowed for participants to join from any device of their choice. Most participants remarked that they were used to online classes (due to the pandemic) and often thought it was convenient. However, other participants remarked that they would have preferred an in-person format:

“It’s alright on Zoom it would have been better in person. More physical. It was convenient but annoying.”

“I’m used to doing classes on Zoom. Zoom is good because it’s convenient. I have a busy schedule with school and work, so it was convenient.”

“The energy is not as it is in person. It’s a whole different type of vibe when we’re in person.”

“Cool to be on Zoom. Probably weird in person because it’s awkward meeting new people and doing something you have never done before. You don’t know what you’re walking into.”

### 3.7. Feedback about Specific Workshop Activities

For each activity type, including Making a Weekly Goal, Let’s Get Moving, Nutrition Knowledge and Skills Building, and Interactive Games, participants ranked how much they enjoyed the activity on a 5-point Likert scale. The average recorded scores were 4.5, 4.5, 4.6, and 4.5, respectively. We present more detailed feedback about each of these activity types below.

### 3.8. Making a Weekly Goal

Participants remarked that it was beneficial and inspirational for them to have and complete weekly goals and realized that they could involve other people in their weekly activities. Participants stated:

“I could hang out with friends or do it at home or with family. It gave me the right mindset. When I wake up, I feel more healthy.”

“The action plans helped me. I knew what to do each day. It was helpful to know/plan what I was doing and for how long.”

### 3.9. Let’s Get Moving

As most participants kept their cameras off during the virtual workshop, it was difficult to gauge their level of engagement with the group exercise activity particularly since, unlike other activity types, this activity did not require participants to speak or type in the chat. We asked participants to reflect on their overall level of engagement, the music in the videos, and the types of exercises displayed. Most participants responded that the music in the exercise videos was acceptable and relevant to youth. Several commented that they appreciated the Let’s Get Moving activity after sitting for most of the session. Others mentioned that their parents or other family members would sometimes pull them away during this portion of the session. A few participants wanted longer and more challenging exercise videos and instead followed their own workouts during this time. Participants mentioned:

“It was a good way to stay active and my uncle sometimes joined. It was more of a reason to exercise.”

“Sometimes I didn’t do it, sometimes I did. Sometimes I worked with my grandma or uncle. [The exercises] were not too hard, not too easy. Sometimes I made my own exercise. The [exercises] with the chair I would change and do like a burpee.”

“I didn’t follow the videos; I just did my own things. The videos didn’t look as intense. I wanted to do more intense workouts to get my body moving. When I first did it was ok but I liked my own thing better. I would change to more HIIT [high intensity interval training] cardio.”

### 3.10. Nutrition Knowledge and Skills Building

Each week, the workshop included activities focused on building participant knowledge and practicing important nutrition-related skills (e.g., label reading, plate planning, and portion control). During our feedback session, participants discussed how these activities were valuable to their understanding of healthy eating and how they shared information they learned with friends and family members. Participants commented:

“Now I know what to do when I get something. Before I was in the store, I didn’t care about the label, and I just drank or ate it. Sometimes I get Arizona so now I put it aside…Sometimes I remember to drink half and give the rest away.”

“…Helped me fix my eating habits, know what to eat and how much to eat and how much to save for later.”

“My grandma doesn’t know the way to plate a healthy plate, but I can tell her and [she] understands.”

“I didn’t know about it until this program. Every time I look at the plate at school cafeterias it’s like they are trying to lecture me. But now I know the reason that it’s there I can tolerate it.”

“I’m confident in using this method. I started eating less fats. I use this to measure out how I eat.”

### 3.11. Games

Most participants found the games to be enjoyable and informative, liked being on teams and working with other participants, enjoyed the competitive aspect, perceived that the games helped participants become more comfortable with each other, and appreciated that they were challenged to remember important concepts they had learned:

“I like brainstorming and competing. The first day was quiet but as soon as we got used to [the sessions]… it was educational but we were having fun.”

### 3.12. Zoom Breakout Rooms

The in-person workshop included small group activities to foster a sense of community and facilitate more intimate group discussions (especially about potentially sensitive topics such as difficult emotions). For the virtual program, we utilized breakout rooms to cultivate this same sense of teamwork and camaraderie. Participants’ reflections on their experiences in these small groups seemed to depend on who was present in the breakout room and the dynamics between the participants. Some mentioned that the breakout rooms were quiet, and it was hard to know if people were there and attentive, especially with cameras turned off. Other participants remarked that the breakout rooms were fun and became more interactive once participants became more familiar with each other as the weeks progressed. Participants stated:

“It was fun in the breakout rooms. I talked more with my peers. We got the chance to hear each other’s thoughts. After week 3 or 4 it was kind of always talkative. It depends who I was with [in the room].”

“At the beginning we were shy. But after we knew each other, we started brainstorming more—it was a process to confide in each other. Week 5/6 (like halfway) we became more comfortable…At the beginning I didn’t want to talk. It was helpful to keep the conversation. The team leader should pump up [the breakout room].”

“I didn’t like them at all unless you were with the peer leader because it was awkward, and you didn’t know anybody. Make sure that one peer leader is always there. Tell us if we don’t do the work we get points taken off.”

### 3.13. Using the Camera on Zoom

Many participants chose not to use the camera function on Zoom. Some reasons cited for staying off camera included technical issues, not wanting to look at themselves or having other people look at them, distractions from family members, or other issues in the environment where the participant was located. Participants commented:

“I use my profile photo and it’s somewhat what I look like. I don’t like looking at myself in the camera or being too prideful. I guess I have low self-esteem.”

“I hate the camera. I couldn’t come on camera because I was always in public places/camera issues. But it does bring people together and makes you feel like you’re not being ignored. A compromise: maybe every week change the angle of the camera and then at the end have your face on camera.”

## 4. Discussion

The current study examined the feasibility and acceptability of a virtual group-based peer-led diabetes prevention workshop for at-risk prediabetic adolescents. We successfully adapted our workshop for virtual delivery on Zoom in collaboration with our community partners (including youth). The results of our study demonstrate that it is feasible to implement this type of program with youth via a videoconferencing modality. We were able to deliver the same workshop content (topics such as label reading, plate planning, portion control, strategies to increase physical activity, and coping with eating triggers and social pressures) and behavioral skills development using a variety of features on Zoom (e.g., screen share, annotation, reactions, chat box, and breakout rooms). The virtual program was also accepted among participants who reported that they gained knowledge related to healthy eating and active living, developed important skills such as goal setting and behavior tracking, and enjoyed the interactive, engaging content (rated the main workshop activities 4.5 out of 5).

Virtual prevention programs such as ours, if proven to positively impact behavioral and clinical outcomes, have the potential to benefit other youth, including those that come from similar socio-demographic backgrounds that are disproportionately impacted by diabetes. We intentionally included topics to address social determinants of health that led to such disparities, such as access to and affordability of healthy food and physical activity and cultural/social contexts [59]. Additionally, given the increased prevalence of behaviors such as unhealthy eating and increased sedentary activity among youth during the COVID-19 pandemic, and the subsequent weight gain and increased diabetes risk that result from these behaviors, it is more urgent than ever to develop innovative diabetes prevention strategies. In addition, the COVID-19 pandemic has made it necessary to consider how to support at-risk youth in the context of lockdowns and social distancing. Virtual prevention programs such as ours can play a crucial role in bridging these gaps by allowing individuals to participate in programs safely and conveniently without having to travel and gather. However, there are also potential drawbacks to consider. Participants in our study were able to use a variety of devices to join the workshop, including desktop computers, laptops, tablets, or smartphones. While teens from lower-income households are just as likely to own a smartphone as wealthier teens and more likely to use their phone to access social networks and health information [62], studies have also pointed to the possibility of inequities that can result from reliance on digital tools for youth education [63,64]. Thus, we must consider socioeconomic challenges, availability of privacy and space to participate in programs at home, comfort with technology, and access to devices and reliable internet services as programs such as ours expand and are disseminated more broadly. There are also privacy concerns that have been emerging in the digital realm that should be considered [65,66].

Our study is one of the first focused on development and implementation of a virtual diabetes prevention program for prediabetic youth. The majority of the literature regarding virtual programs published to date has focused on adults. In contrast to our study, online diabetes prevention programs for adults have been delivered primarily through asynchronous methods. These programs also employ a combination of remote peer support, coaches, or family members monitoring progress virtually through third party smartphone applications [32,47,48,49,50]. Similar to these types of virtual adult programs [40,41,42,46], our intervention largely focuses on lifestyle factors such as healthy eating and being physically active. In addition, we address topics such as coping with stress/difficult emotions, body image, healthy vs. unhealthy weight control behaviors, the local neighborhood environment, cost/affordability, marketing, media, communication skills, mindfulness, and advocacy. As in the adult programs, we emphasize important skills such as goal setting and problem solving. We also intentionally incorporated other interactive components, including games, role plays, individual and team-based competitions, and group exercise with videos and live instruction, to better engage our younger target population.

While there is not published literature about virtual youth diabetes prevention programs, we did find information about virtual youth interventions focused on other chronic diseases [54,55]. Similar to our program, these interventions were based on behavior change models such as the transtheoretical model and the health belief model [67,68]. However, the focus for such interventions and the delivery methods differed from ours. For instance, most studies examining asthma focused on medication adherence and symptom control [69,70]. Educational content for these interventions was also delivered asynchronously though pre-created video demonstrations from trained staff, websites, and/or chat rooms in contrast to our study, which employed peer educators who delivered content through video conferencing in real-time. The only other youth chronic disease interventions we found that used live video conferencing methods were interventions focused on providing cognitive behavioral therapy for children with chronic conditions and mental health conditions such as anxiety [71,72,73].

Virtual youth lifestyle modification interventions have shown promise in improving lifestyle behaviors and achieving weight loss [55]. Many of these interventions utilized a combination of face-to-face delivery along with mHealth (social media, text messaging, mobile apps, and websites) [21,22,23] and some have used gamification combined with personalized fitness coaches to promote physical activity [74,75,76]. Although we did not use gaming software or devices in our intervention, we did include interactive games to promote behavior modification. The only other study we could find that included remote education and counseling about lifestyle behaviors through video conferencing focused on adolescents with intellectual disabilities and found no differences in outcomes for participants who received the program in-person or virtually [77,78].

Virtual engagement is often difficult, especially if the group has never met in-person and participants have not previously developed relationships with the peer leaders and others in the group. There were times when there was limited participation from the participants, which often required peer leaders to offer ideas or get conversations started. In addition, many teens stayed off camera most of the time either because they were uncomfortable in the virtual setting or because they were used to being off camera during remote learning during the pandemic. Thus, in contrast to the in-person workshop where peer leaders assessed whether participants were paying attention through eye contact and body language cues, we sometimes had a hard time assessing the level of engagement during the virtual sessions. Regardless, our choice of Zoom as our virtual platform allowed us to utilize several features to engage with youth in ways that were acceptable and helpful to them. For example, teens were able to communicate with us directly (either on camera or off) and through the chat box feature if that was more comfortable for them. We also used reactions such as “applause” or “celebration” to provide positive reinforcement and motivation and asked participants to use reactions or emojis to share their feelings. We encouraged teens to come on camera and/or “unmute” when they shared personal information and to reflect something about themselves (e.g., their favorite band or a photo of their pet) by updating their profile pictures. In addition, we provided binders to participants that included the most important information presented in sessions so they could view the material easily if they used their phone to join the workshop, look for answers to questions during games, take notes, and refer back to the information in between sessions and after completion of the workshop. We also used appealing graphics and colorful slides throughout the workshop to heighten interest and engagement. We noted that all these strategies led to increased comfort and engagement over time, with more willingness to come on camera or speak out loud as the workshop progressed.

There were several advantages and challenges to using the breakout room function on Zoom. Breakout rooms allowed for teams to strategize and brainstorm answers during games and created more intimate spaces with smaller groups to encourage honest conversations, especially about more sensitive topics. However, some activities that we planned to conduct in breakout rooms were instead conducted in the main room when fewer participants were present. There are also some logistical considerations when using breakout rooms, including the time and effort needed for the peer leader to create and monitor the rooms and for the participants to enter and exit the rooms. We also noticed that participants sometimes dropped out of the session during breakout room transitions either intentionally due to decreased engagement or unintentionally due to clicking on the wrong button or other technical issues.

We noted several differences when reflecting on the delivery of our intervention virtually versus in-person. For instance, the in-person workshops required significant preparation from the peer leaders, including arriving early to gather materials such as flip charts and exercise mats and setting up and cleaning up the room. In contrast, the virtual workshops eliminated the need for in-person setup, as charts were created via the PowerPoint slides, the whiteboard, and the annotate function on Zoom. In-person workshops also required peer leaders to walk around the room and assist participants during certain activities. During the virtual workshop the peer leaders could instead assist participants via the chat box or offline. Some activities conducted in-person, including certain games, were difficult to adapt for virtual delivery, and we had to develop alternative activities to reflect the same content. Furthermore, we encountered some challenges with the group exercise component of our program when delivered virtually. Unlike during the in-person workshop, peer leaders could not assess participation or proper completion of the exercises as most of the participants were not on camera. Additionally, not every participant could access every Zoom function (for example, some could not annotate), making it difficult for them to participate in certain activities. Finally, there was a learning curve for peer leaders and participants to utilize certain Zoom features. Peer leaders had to become comfortable with multitasking (i.e., sharing their screen, annotating, creating teams and breakout rooms, keeping track of points, and monitoring the chat box while also referring to their peer leader manual in a separate window to conduct the workshop activities). Regardless, with practice and teamwork, the leaders were able to effectively deliver the curriculum as intended and became more comfortable as the workshop progressed.

The limitations of our study include a small sample size, as this was a pilot study to assess the feasibility and acceptability of a virtual intervention. Our sample was skewed towards younger teens (aged 13–15) with fewer participants who were 16 years or older. Although we received consent from parents/caregivers of 14 prediabetic adolescents, only half could attend the program due to scheduling conflicts, as we only offered the workshop once a week at a specific day/time for this pilot. We anticipate higher participation rates in the future when we are able to offer more options. Another limitation is that we recruited patients from one urban clinic (which serves racially/ethnically diverse families, largely Hispanic/Latino and Black, from East Harlem and the South Bronx), and findings are not generalizable to youth from other settings and backgrounds. Furthermore, the current study used a qualitative approach rather than quantitative assessment of clinical outcomes such as weight or hemoglobin A1c. Despite this, as this was one of the first studies to pilot a virtual diabetes prevention program among at-risk youth, the information we learned about the adaptation of an in-person program, what was feasible to implement virtually, and what aspects of the program youth engaged with and enjoyed is still valuable.

### Future Directions

This study provided preliminary insights into potentially effective strategies for the implementation of a virtual diabetes prevention program for adolescents. We are currently implementing the virtual program with cohorts of prediabetic youth (including younger and older teens) from our community and examining the impact of the program on behavioral and clinical outcomes. We also have to consider whether a hybrid model in which there is some combination of virtual and in-person interactions would be beneficial. This type of model would allow us to balance the benefits of virtual delivery (less need for space, convenience, and potential savings in cost and time) with the energy and relationships that are sometimes better fostered in-person. Further, there may need to be some type of vetting process to ensure that the teens who participate are engaged with the topic and will actively take part in the program. Finally, there are concerns about retention rates for digital interventions, which may pose biases through loss to follow up and/or attrition [79,80]. Future studies should thus consider the best ways to recruit and retain youth in virtual interventions.

## 5. Conclusions

Our study resulted in the adaptation of an in-person lifestyle intervention for prediabetic youth to a virtual format. We demonstrated feasibility and acceptability of live video conferencing for delivery of our diabetes prevention workshop. Our study is novel in that it focused on youth, diabetes, peer education, and delivery of group-based workshops in real-time rather than through asynchronously delivered content. We are continuing to implement and evaluate our program to assess its impact on lifestyle behaviors and clinical outcomes. If proven successful, this model may be used and disseminated to similar populations and communities to address diabetes-related disparities.

## Figures and Tables

**Table 1:** Adaptations for virtual program delivery.

In Person	Virtual Adaptation	Screen Shot
Charts with key workshop content made by leaders using flip charts based on samples in peer leader binder.	Create PowerPoint slides with all charts for hard copy and electronic binders for peer leaders and participants. Share slides in real-time during the virtual workshop session using screen share.	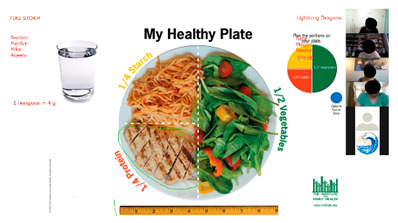
Write on flip charts for brainstorming, problem-solving, and other activities.	Utilize the “white-board” and annotation functions on Zoom to document ideas shared.	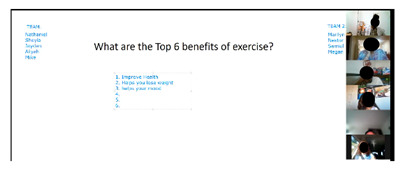
To set weekly goals, peer leaders walk around the room and assist participants with making their goal if needed. They then ask for a volunteer and go around the room to share plans.	Ask for a volunteer and then call on participants based on the order they are listed in the Zoom participant list. Participants may also choose to share their goal in the chat box with peer leaders prompting them for any information that is missing to make the goal as specific as possible or encouraging them to modify their goal so that they have a high confidence level that they can complete it.	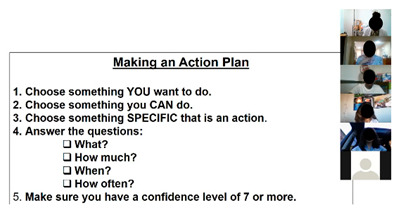
Bring in, and share, food and drink labels.	Create PowerPoint slides with images of relevant nutrition labels for hard copy and electronic binders. Use screen share to share during workshop sessions.	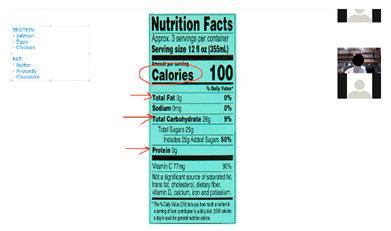
“Let’s get moving” group exercise activity.	Share premade exercise videos using screen share and then spotlight a peer leader to model the exercises in real-time on camera to increase motivation and support. Plan modifications not only for different ability levels but also for teens with limited space to exercise. Create a library of videos for participants to use any time.	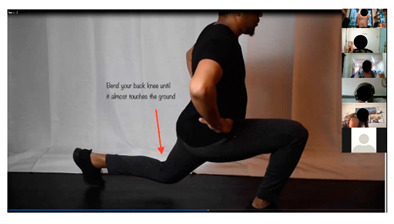
Use buzzer or hit the table when participants know answers to questions during interactive games.	Ask participants to type answers in the chat box to keep track of who gave the correct answer first and award points.	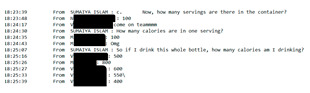
Divide the group into teams for games and small group activities.	Assign teams to breakout rooms for games and small group activities supervised by peer leaders and study staff.	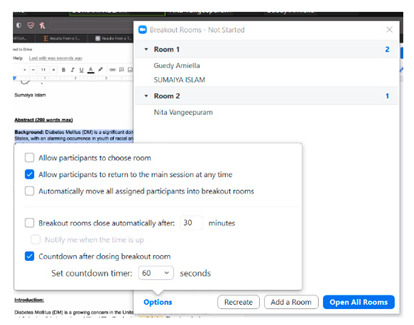

**Table 2:** Characteristics of consented participants.

Gender	Age	Race/Ethnicity
Male	15	Asian
Male	13	Latino
Male	18	Latino
Female	13	Latino
Female	14	Latino
Female	15	Black
Male	14	Latino
Male	18	Latino
Male	15	Black
Male	13	Black
Female	17	Black
Female	13	Latino
Male	14	Latino
Male	13	Latino

## Data Availability

The data presented in this study are available on request from the corresponding author. The data are not publicly available due to protection of privacy and compliance with the Health Insurance Portability and Accountability Act (HIPAA).
